# SiteHopper - a unique tool for binding site comparison

**DOI:** 10.1186/1758-2946-6-S1-P57

**Published:** 2014-03-11

**Authors:** Jose Batista, Paul CD Hawkins, Robert Tolbert, Matthew T Geballe

**Affiliations:** 1 OpenEye Scientific Software, Cologne, 50672, Germany; 2 OpenEye Scientific Software, 9 Bisbee Court, Suite D, Santa Fe, NM, 87508, USA

## 

Knowledge of and information about protein binding sites has become increasingly important in the drug discovery process and not just for molecular biologists [1]. By comparing binding sites within and across protein families, relevant details about the functionality and selectivity of a target protein can be extracted leading to useful insights for the development of new ligands [2]. SiteHopper provides a powerful alternative method to the traditional use of sequence alignment for this purpose. Using OpenEye's Shape [3] and Spicoli toolkits [4], SiteHopper quickly calculates a 3D shape representation of the active site colored by the chemical properties of the protein residues defining the active site. These active site representations can be rapidly aligned and assessed for shape and chemistry similarity. As SiteHopper is built on the OpenEye toolkits, it is highly flexible and customizable for a variety of end-uses. In this presentation, the methodology behind SiteHopper will be introduced and multiple relevant applications will be shown.
